# Post-synthesis nanostructuration of BSA-Capsaicin nanoparticles generated by sucrose excipient

**DOI:** 10.1038/s41598-021-87241-8

**Published:** 2021-04-06

**Authors:** Ramón Carriles, Laura E. Zavala-García, Sofía Nava-Coronel, Alejandro Sánchez-Arreguín, Mercedes G. López, Lino Sánchez-Segura

**Affiliations:** 1 División de Fotónica, Centro de Investigaciones en Óptica, A.C, Loma del Bosque 115, León, Guanajuato 37150 México; 2 Departamento de Ingeniería Genética, Centro de Investigación y de Estudios Avanzados del Instituto Politécnico Nacional, Unidad Irapuato, Km. 9.6 Libramiento Norte, Carretera Irapuato-León, Guanajuato, 36824 México; 3 Departamento de Ingeniería en Nanotecnología, Instituto Tecnológico Superior de Ciudad Hidalgo, Av. Ing. Carlos Rojas Gutiérrez 2120, Ciudad Hidalgo, Michoacán 61100 México; 4 Departamento de Bioquímica y Biotecnología, Centro de Investigación y de Estudios Avanzados del Instituto Politécnico Nacional, Unidad Irapuato, Km. 9.6, Libramiento Norte, Carretera Irapuato-León, Guanajuato, 36824 México

**Keywords:** Nanobiotechnology, Nanoscale materials

## Abstract

In the pharmaceutical industry nano-hydrocolloid systems frequently coalesce or present nanoparticle aggregation after a long storage periods. Besides, the lyophilization process used to dry nanoparticles (NPs) produces loss of their original properties after dispersion. In this work we evaluated the effect on morphology and physicochemical properties of different protective excipients during drying of bovine serum albumin (BSA) NPs loaded with different concentrations of capsaicin. Capsaicin concentrations of 0, 812, 1625, 2437, and 3250 µg mL^−1^ were used; subsequently, NPs were dried with deionized water (DW), NaCl (DN), sucrose (DS), and not dried (ND). We found that ND, DW, and DN treatments showed a negative effect on the NPs properties; while, DS reduced the aggregation and produced the formation of isolated nanoparticles at higher concentrations of capsaicin (3250 µg mL^−1^), improving their circular shape, morphometrical parameters, and ζ-potential. The stability of the BSA-capsaicin NPs was associated to complex capsaicin/amino acid/water, in which GLY/GLN, ALA/HIS, ARG, THR, TYR, and Iso/CYS amino acids are involved in the restructuration of capsaicin molecules into the surface of nanoparticles during the drying process. The secondary nanostructuration in the post-synthesis stage can improve the molecular stability of the particles and the capacity of entrapping hydrophobic drugs, like capsaicin.

## Introduction

Nanotechnology has been used with great impact in the pharmaceutical industry. The encapsulation of the active formula into nanocarriers produces an increment of drug bioavailability in the target cells and tissues and reduces adverse effects^1^. Among these technologies, special attention has been dedicated to the study of bovine (BSA) and human (HSA) serum albumins nanoparticle (NP) production. These biopolymers have been used to encapsulate several drugs; for example salicylic acid, pranoprofen, antineoplastic agents (curcumin, vincristine, vinorelbine (Navelbine IV) and vinblastine), and chemotherapy drugs such as 5-fluorouracil (5-FU), paclitaxel (Abraxane)^2–7^. In agricultural science, albumin has been used to potentiate antimicrobial effects, load extra cellular chitinase, and to encapsulate hydrophobic secondary metabolites of plants to inhibit phytopathogens growth^8–10^.

The mechanism of albumin transformation into nanoparticles has been greatly studied^11^. It has been found that the main parameters that affect the synthesis are initial protein concentration, temperature, pH, glutaraldehyde concentration, agitation speed, rate of addition of crosslink/desolvation agent, and drug load^10,12–14^. Only few studies have focused on the description of post-synthesis stages, i.e. hydrocolloid stability, storage, effect of the excipient during dried freeze, and changes of physicochemical properties after redispersion.

Nano-hydrocolloidal systems suffer from NP aggregation after long storage periods, limiting their use for drug delivery^1,4^; shelf life can be improved by removing the water contained in them. Lyophilization is commonly employed to stabilize different types of NPs^1,15,16^; however, after lyophilization and dispersion, most nanoparticles do not maintain their original properties. Anhorn et al.^1^ found that the sucrose excipient at 3% in freeze-drying of HSA nanoparticles improves long-term storage stability with respect to the particle diameter and polydispersity after reconstitution; while, Kim et al.^2^ found that the freeze-drying with water maintained a stable aspect of the lyophilized cake. Interestingly, both studies theorize about the negative influence of lyophilization in NP stability; however, in neither study the morphology or any physicochemical parameters of the NPs were evaluated.

Capsaicin [(E)-*N*-(4-hydroxy-3-methoxyphenyl)methyl-8-methylnon-6-enamide], the metabolite found in pungent chili peppers (*Capsicum *spp.), has been used in novel biotechnological applications entrapped in BSA nanoparticles^10,14,17^. Nonetheless, the effects of drying conditions in BSA structured NPs and of different concentrations of capsaicin in nano-hydrocolloid stabilization have not been studied. In this work, we evaluated the influence of protective excipients during the drying process with deionized water (DW), NaCl (DN), and sucrose (DS) solutions and not dried (ND) treatment of bovine serum albumin nanoparticles loaded with different capsaicin concentrations, and the effect in the morphological and physicochemical parameters after redispersion of the NPs.

## Results and discussion

### Effect of drying on yield and efficiency of BSA-capsaicin nanoparticles

**Figure 1:**
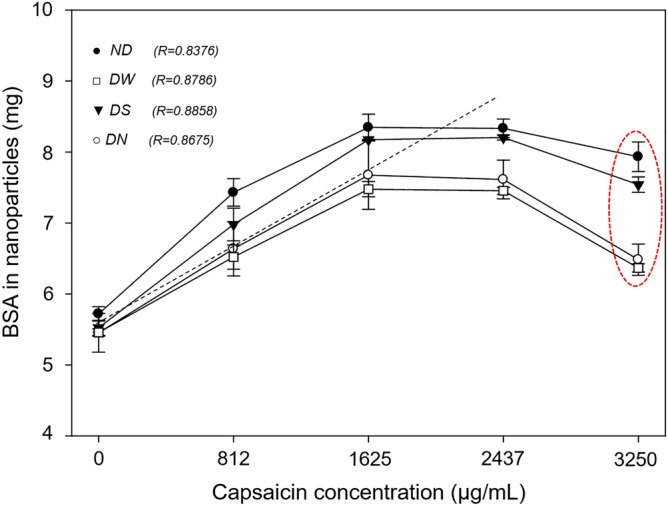
Effect of capsaicin concentration in the quantified BSA in nanoparticles subjected to drying treatments: not dried (ND), dried with water (DW), dried with sucrose (DS), and dried with NaCl (DN). All treatments showed a linear tendency for low concentrations. Values are means of three experimental replicates ± standard deviations.

The ND treatment showed a positive correlation (R = 0.8376) between the transformation of native BSA into nanoparticles and drug concentration during the coacervation process (Fig. 1, full circles); a similar linear tendency was reported by Sánchez-Segura et al*.*^14^ and Sánchez-Arreguin et al*.*^10^ while the nanoparticle yield showed the highest values at 1625 and 2438 µg mL^−1^ of capsaicin (91.8% and 91.7%, respectively), the yield decreased for 3250 µg mL^−1^ (Table 1 and red dashed ellipse in Fig. 1). This reduction is due to saturation of the amino acids responsible for entrapping the capsaicin. This result confirms previous observations that the increment of capsaicin concentration affects the transformation of native BSA into nanoparticles^10^. The interaction of BSA molecules with an hydrophobic drug, improved the yield of nanoparticles respect to simple nanostructuration of BSA in which the yield reached between 68 and 70%^7^. On the other hand, drying treatments with different excipients showed a slightly higher correlation between BSA transformed into NPs and the increment in the concentration of capsaicin. No additional BSA or capsaicin were supplemented during the drying process. The correlation factors for different treatments were R = 0.8786 for DW (Fig. 1, empty squares), R = 0.8675 for DN (Fig. 1, empty circles) and R = 0.8858 for DS (Fig. 1, full triangles). On the other hand, the DS treatment showed more affinity of the BSA to capsaicin molecules at a low concentration of capsaicin (812 and 1625 µg mL^−1^, see Table 1). The maximal values in the ratio BSA/capsaicin at higher concentrations (2437 and 3250 µg mL^−1^, see Table 1) were found in ND and DW, respectively. However, the DS treatment maintains a high molecular affinity (see Table 1). The yield values for all drying treatments showed a slight decrement as compared to the ND treatment; however, the DS treatment showed a lower loss of BSA during the drying process. The nanoparticle yield of the DS treatment showed its highest values, 89.9% ± 0.1 and 90.2% ± 0.4, at 1625 and 2438 µg mL^−1^ of capsaicin concentration, respectively; but at 3250 µg mL^−1^ the yield decreased (Table 1).

**Table 1:** Nanoparticle yield, entrapment efficiency and ratio BSA/Capsaicin.

Capsaicin concentration (µg mL^−1^)	BSA nanoparticles yield (%)^a^			
	ND	DW	DS	DN
0	62.9 ± 1.1	60.0 ± 0.6	60.8 ± 1.2	60.0 ± 3.0
812	81.7 ± 2.1	71.7 ± 1.9	76.8 ± 2.5	73.0 ± 4.2
1625	91.8 ± 2.0	82.2 ± 1.2	89.9 ± 0.1	84.4 ± 5.3
2437	91.7 ± 1.4	82.0 ± 0.6	90.2 ± 0.4	83.7 ± 3.0
3250	87.3 ± 2.3	70.0 ± 0.6	82.9 ± 1.2	71.3 ± 2.4

Capsaicin concentration (µg mL^−1^)	Encapsulated efficiency (%)^b^			
	ND	DW	DS	DN
0	00.0 ± 0.0	00.0 ± 0.0	00.0 ± 0.0	00.0 ± 0.0
812	49.0 ± 0.3	44.3 ± 0.3	49.1 ± 0.8	46.3 ± 0.3
1625	59.0 ± 2.8	46.0 ± 0.5	58.5 ± 1.9	46.3 ± 3.7
2437	75.6 ± 3.3	60.0 ± 1.6	72.7 ± 2.2	64.4 ± 4.7
3250	55.8 ± 0.8	46.2 ± 0.6	55.4 ± 1.5	48.8 ± 3.3

Capsaicin concentration (µg mL^−1^)	Ratio BSA/Capsaicin			
	ND	DW	DS	DN
0	0.0	0.0	0.0	0.0
812	4.7	4.9	5.0	4.8
1625	10.0	8.6	10.1	8.7
2437	19.2	17.9	18.8	17.1
3250	19.9	21.3	20.8	20.5

^a,b^n = 3 ± SD.

The post-nanostructuration effect triggered by the excipients and drying process has not been described yet for BSA nanoparticles; however, biopolymer reorganization by temperature and chemical stimulus was studied previously on nanoparticles. Yang et al.^18^ found that nanoparticles formed by blocks of bis(pyrene)-Lys-Leu-Val-Phe-Phe-Gly-polyethylene glycol (BP-KLVFFG-PEG, BKP) and hydrophilic polyethylene glycol (PEG) showed a spontaneous reorganization of structure. Probably this change was due to the strong hydrophobic interactions when the BKP self-assemblies in water. A similar effect was observed by Costa et al.^19^; they found that microcapsules of Chitosan/ELRs (biomimetic elastin-like recombiner) showed stimuli-responsive effect caused by different solvent temperatures. The synergy of these stimuli produces significant changes probably as a result of a layer rearrangement of chitosan that allowed improving the control of the permeability of these multilayer systems.

**Figure 2:**
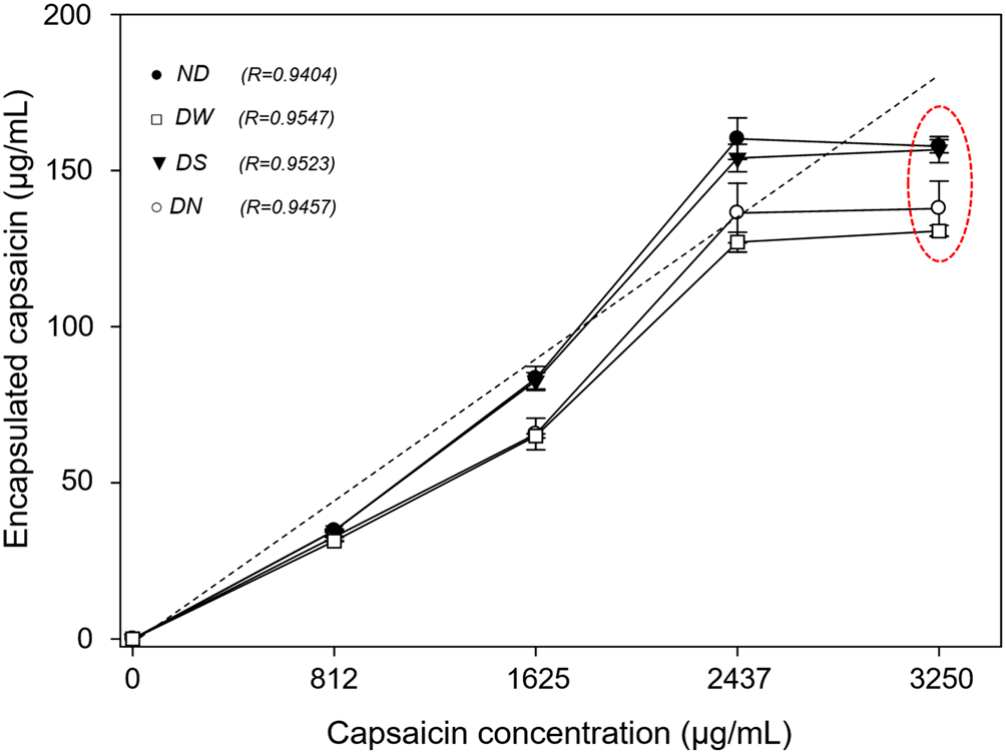
Encapsulated capsaicin in nanoparticles as a function of initial concentration. The quantified capsaicin showed a linear tendency followed by a plateau for all drying treatments. Values are means of three experimental replicates ± standard deviations.

On the other hand, the encapsulated capsaicin recovered from ND treated samples showed a linear correlation (R = 0.9404) with respect to capsaicin concentration, 0, 812, 1625, 2437, and 3250 μg mL^−1^ (Fig. 2, full circles). During the coacervation process, it was also possible to observe the effect of BSA saturation (Fig. 2, red ellipse). Drying treatments with water, sucrose, and NaCl excipients showed similar linear trends with R = 0.9547, R = 0.9523, and R = 0.9457, respectively (Fig. 2). The correlation showed slight changes, probably due to the amino acid-capsaicin interaction in the nanoparticles being affected by the drying process, it is possible that low quantities of capsaicin were delivered from the NP surface to the supernatant during reconstitution of the nanoparticles. De Freitas et al*.*^20^ reported that BSA-capsaicin nanoparticles stored at freezing conditions (− 4 °C) showed a lower drug release reaching 7% of capsaicin entrapped, compared to NPs stored at room temperature that reached 31% and NPs stored under refrigeration (4 °C) showed 18% of released capsaicin after 3 months.

Regarding the Encapsulated Efficiency percentage (*EE%*), it showed large changes between different drying treatments (Table 1); actually, the *EE%* was more affected by the drying process than any other variables reported in this work. A capsaicin concentration of 2437 μg mL^−1^ produced the highest *EE%* for all four drying treatments; 75.6 ± 3.3% for ND, 60.0 ± 1.6% for DW, 64.4 ± 4.7% for DN, and 72.7 ± 2.2% for DS (Table 1). Therefore, the highest capsaicin loss occurred when deionized water or NaCl were used as protective excipients in the drying process. It has been reported that albumin NPs suffer loss of loaded drug impacting negatively the pharmacokinetics, drug delivery, and therapeutic efficacy^21^. This has been partially resolved by using mannitol, sucrose, and trehalose excipients during the freeze-drying of BSA nanoparticles, allowing a reduced loss of drug load of up to 1%^1^.

### Structural changes of BSA-capsaicin nanoparticles after drying treatments

**Figure 3:**
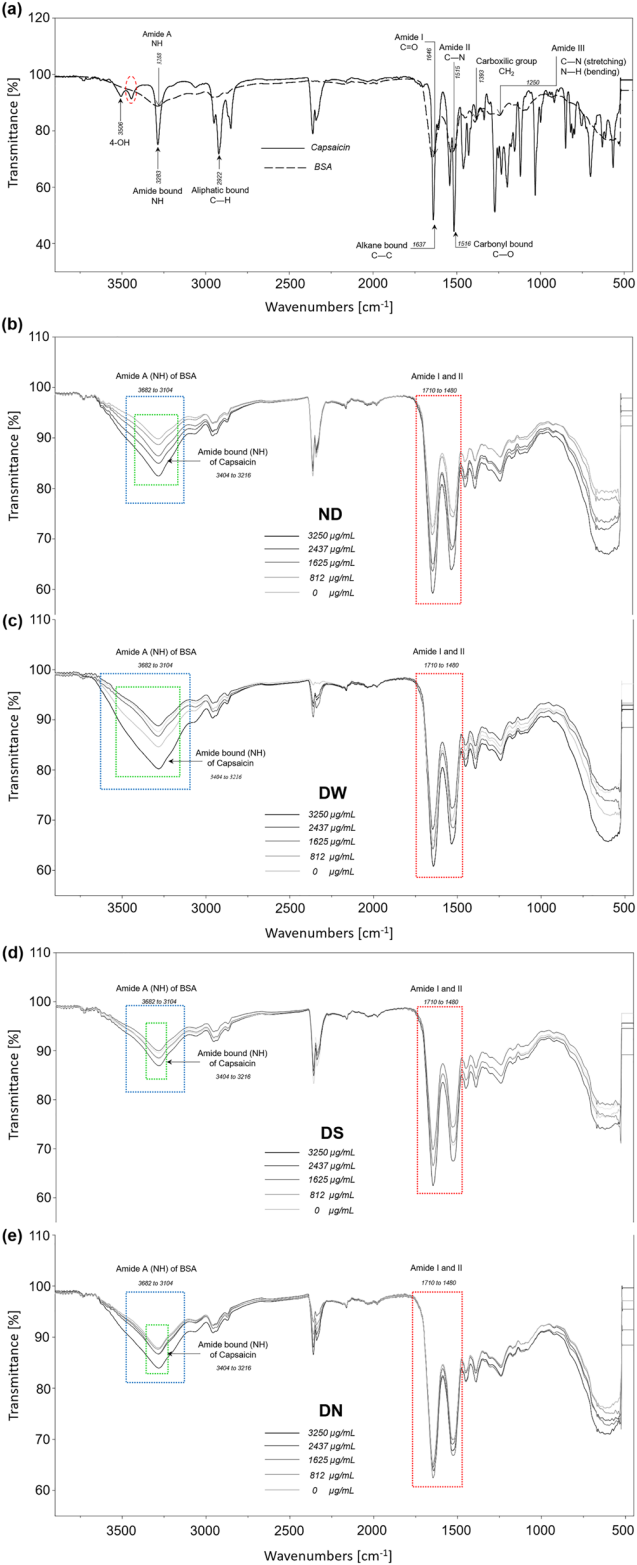
Molecular changes of the BSA-capsaicin nanoparticles after drying treatments with several excipients and the effect of the increment of capsaicin in the nanostructuration. (**a**) FTIR spectrum of native BSA (dotted line) and pure capsaicin (solid line); the scanning spectral range was between 4500 and 400 cm^−1^. (**b**) FTIR spectral characterization of not dried treatment (ND) of BSA-capsaicin nanoparticles at 0, 812, 1625, 2437 and 3250 µg mL^−1^, rectangles show the main change in amide A and bound amide of capsaicin. (**c**) NPs dried with water (DW), rectangles show changes in alkane and carbonyl bonds, functional groups associated with molecular water. (**d**) NPs dried with sucrose (DS), spectra did not show deformation. (**e**) NPs dried with NaCl (DN), spectra showed a low deformation in treatments from 0 to 2437 μg mL^−1^ of capsaicin concentration, at 3250 μg mL^−1^ an increase of transmitted signal of the capsaicin was observed.

Reported Fourier Transform Infra Red (FTIR) spectra from pure capsaicin and native BSA^10,22,23^ were compared against the spectra from our samples. The solid line in Fig. 3a shows the FTIR spectrum from our native capsaicin; the phenolic 4-OH group was assigned to the peak at 3506 cm^−1^, this resonance showed slight changes with respect to previous reports^10,24^. In this work we found a peak at 3443 cm^−1^ probably due to photochemical oxidation of capsaicin during analysis (Fig. 3a, red ellipse). The 4-OH of capsaicin, show high reactivity and decomposition during analysis of the chemical structure by NMR, HPLC, and FTIR^25^. A third peak was found at 3283 cm^−1^ and corresponds to the amide stretching bond N–H of the capsaicin molecule. The peak at 2922 cm^−1^ was associated to the aliphatic bond C–H stretching vibration. Finally, the resonances at 1637 and 1516 cm^−1^ were assigned to the C–C and C–O stretching vibrations, according to Leela et al*.*^26^.

The dashed line in Fig. 3a, shows the spectrum for native BSA; similar profiles have been observed by Bronze-Uhle et al*.*^3^ and Sánchez-Arreguin et al.^10^. The N–H functional group associated to amide A showed a broad stretching with a maximal peak at 3288 cm^−1^. The second maximal peak, found at 1646 cm^−1^, corresponds to a stretching of the carbonyl group (C=O) of amide I. The subsequent regions showed some intense peaks associated to functional groups with double molecular behavior. C–N stretching and N–H bending of amide II was identified at 1515 cm^−1^. The CH_2_ bending groups were associated to the peak at 1393 cm^−1^, while the vibrance at 1250 cm^−1^ was correlated to C–N stretching and N–H bending in amide III.

All treatments (ND, DW, DN, and DS) and formulations of capsaicin (0, 812, 1625, 2437, and 3250 μg mL^−1^) showed deformations of the peaks on the region from 3682 to 3104 cm^−1^; this spectral region corresponds to the N–H amide A of the BSA, and overlaps with the 4-OH group and NH amide bond of capsaicin (see blue rectangles in Fig. 3b–e). For all treatments with capsaicin concentration of 3250 μg mL^−1^ the NH amide bond signal of pure capsaicin (N–H stretching) in the region from 3404 to 3216 cm^−1^ increased as shown by the black line in the green rectangle of Fig. 3b–e. The spectral analysis of each treatment serves to identify possible structural changes in the nanoparticles after the drying procedure. The DW and DN treatments at 3250 μg mL^−1^ showed an intense peak deformation in the 3404 to 3216 cm^−1^ region as displayed by the black lines in the blue rectangles in Fig. 3c,e, respectively. These perturbations were probably due to the instability of the capsaicin during the drying process; deionized water and NaCl excipients showed no protection capacity against changes generated in the interaction of the amino acid of albumin/capsaicin/water.

As Fig. 3d shows (see blue and green rectangles) the spectral resonances of the overlapping peaks of N–H amide and A-/-NH amide bonds of capsaicin (3404–3216 cm^−1^) for the DS treatment did not show perturbations in intensity and functional groups for all concentrations. This observation suggests the low deformation of the peak and the low distance between bands of transmittance were probably due to the ordered interchange of the amino acid of BSA in the microstructure and the subsequent migration of capsaicin from the core to the surface of the nanoparticles^10,14^. This leads to an homogeneous distribution of capsaicin patches on the surface of NPs dried with sucrose.

The DS treatment showed the best protective effect on the functional groups of amino acids of BSA NPs; a similar effect was observed by Lee and Timasheff^27^ they found that sucrose does not perturb the spectral fingerprint of several proteins during the drying process. The stabilization of proteins by sucrose excipient has been proposed by the formation of polyhydric alcohols that induce a conformational change in some functional groups of proteins, producing milder changes. In this study, during the drying process with sucrose, the capsaicin experienced an equilibrium of repulsive forces between capsaicin and hydrophobic amino acid of BSA, into the nanoparticles. During this process, the restructuration of capsaicin is carried out, and the capsaicin passed from the core to the surface of the nanoparticles; we found this restructuration is less violent when NPs are dried with the sucrose excipient.

The efficiency of excipients in the drying process of NPs has been evaluated through the presence of free H_2_O or molecular water (O–H) content in the FTIR spectrum at 1644 cm^−1^ wavelength^28^. In this work, this region corresponds to the overlapping of amide I and II bands of BSA with the hydrophobic side chain of the capsaicin (1710–1480 cm^−1^), see the red rectangle in Fig. 3b–e. The presence of molecular water was observed in nanoparticles dried with and without water, while in the DS and DN treatments the presence of O–H was not observed. Moreover, a protein–sugar interaction was not observed for the DS treatment; this peak was reported at 1580 cm^−1^, which is ascribed to the H-bond interaction with the carboxylate groups^29^.

### Morphology of nanoparticles

**Figure 4:**
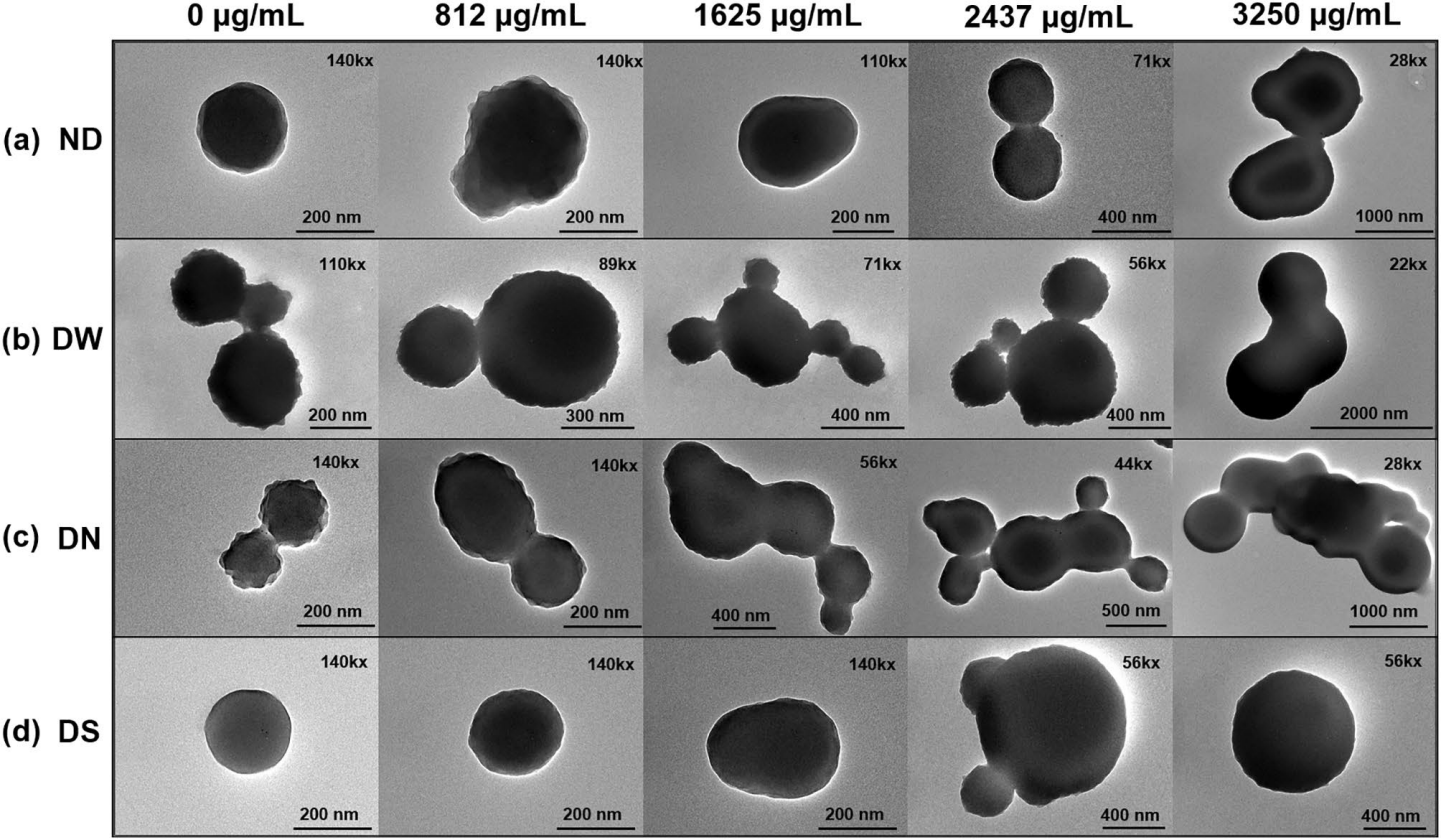
Morphology of BSA-capsaicin nanoparticles. (**a**) Not dried (ND), (**b**) dried with water (DW), (**c**) dried with NaCl (DN), and (**d**) dried with sucrose (DS). Electron micrographs showed a synergy effect between loaded capsaicin and drying procedure. The isolated nanoparticles showed a change of shape, size and, in some cases, showed coalescence. TEM micrographs showed several magnifications (upper right margin) due to the increased NPs size.

Regarding the NPs size, they increased as the concentration of capsaicin increased; this trend has been observed before^10,14,17^. Beside size changes, the TEM images showed morphological changes in the nanoparticles depending on the drying treatment. As shown in Fig. 4a (for more images see Fig. S2 of Supporting Information), the ND treatment showed a transitional change of shape in nanoparticles from circular shape, for 0 µg mL^−1^ of capsaicin concentration, to elliptical shape for 1625 µg mL^−1^. It can also be observed that at higher capsaicin concentrations NP coalescence is more extensive and small aggregates are produced.

The treatment DW produced large particles with an elliptical shape and secondary particles branching from the structure and forming columnar aggregates as seen in Fig. 4b (for more images see Fig. S2 of Supporting Information). It is also noticeable that the surface of the NPs was slightly rougher; a similar effect was observed in BSA-capsaicin nanoparticles reported by De Freitas et al*.*^20^. It is probable that the dual effect of freeze drying and high capsaicin concentrations produced columnar aggregates with quasi-spherical shape.

Among the four treatments, DN resulted in the most negative effects regarding NPs shape. In this drying procedure even the small nanoparticles with 0 µg mL^−1^ of capsaicin fused between them as can be appreciated in Fig. 4c (for more images see Fig. S2 of Supporting Information). Similar coalescence was observed for the other capsaicin concentrations; also it is worth noting that as the drug load increased, the structural complexity of the NPs decreased, probably due to amino acids disassembling and producing an amorphous coagulated protein.

Interestingly, the DS treatment resulted in better morphology even than the ND case. We found that the sucrose at 1 mmol, allowed to protect the circular shape of nanoparticles observed at 0 µg mL^−1^ of capsaicin, see Fig. 4d (for more images see Fig. S2 of Supporting Information) and improved the morphology of the NPs loaded with capsaicin; this was probably because the removal of water molecules increased sucrose concentration leading to the formation of a *sol*, the residual water molecules produced nanofluidic drag (microcirculation phenomena)^30^. This drag induced the separation between NPs due to shear forces or strong capillary forces^27^. Physical separation of NPs produced an improvement in the morphology of the dried nanoparticles with sucrose.

### Morphometric analysis of the nanoparticles

**Figure 5:**
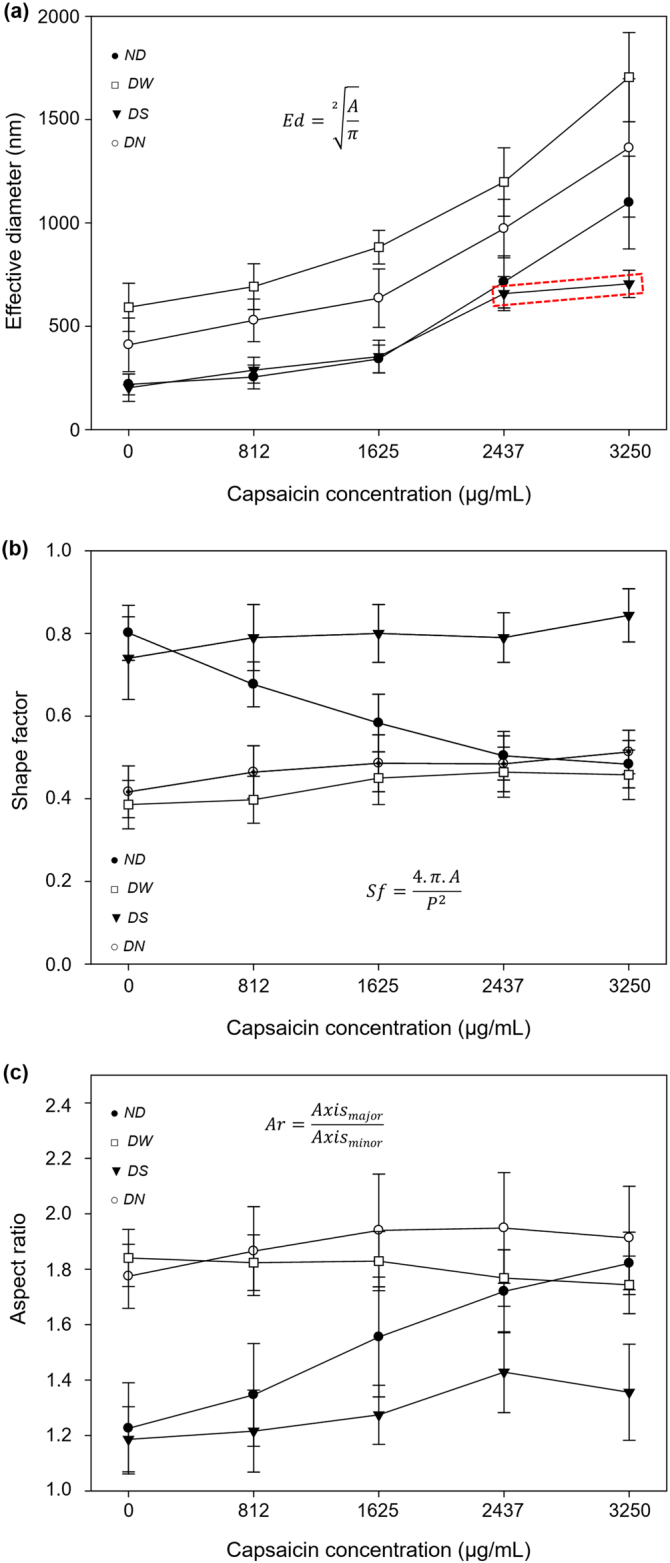
(**a**) Effective diameter parameter of isolated BSA-capsaicin nanoparticles versus capsaicin concentration for different drying procedures. The DS treatment showed a protective effect that allowed to reduce the size of nanoparticles at a high concentration of capsaicin. (**b**) The isolated nanoparticles showed a gradual loss of shape compared with NPs without drying (ND) treatment as a function of initial capsaicin concentration. DW and DN treatments did not show protective effect in any formulation. DS showed a protective effect and increase of the circular shape (*Sf* = 1). The equation of shape factor (*Sf*) is also displayed. (**c**) Aspect ratio of BSA-nanoparticles versus capsaicin concentration. ND treatment resulted in a gradual transition from circular to ellipsoidal shape. DW and DN did not show a protective effect in all formulations. The NS treatment showed a protective effect that reduced the change to elliptical shape at a high concentration of capsaicin (3250 μg mL^−1^). Values are means of three experimental replicates ± standard deviations.

Several studies have reported that the morphometry of nanoparticles changes as a function of the increment of capsaicin during the synthesis of NPs^10,14,17^. In this study, as aforementioned in the morphology analysis section, we observe that the shape of the NPs was affected by the drying process. In order to evaluate this change, TEM images of isolated nanoparticles were analyzed by digital image analysis (DIA). As seen in Fig. 5a all the treatments showed an increment of effective diameter (*Ed*) with increasing drug load. The *Ed* for the ND treatment changed by a factor of 5.0 from 0 to 3250 µg mL^−1^ of capsaicin concentration; for the other treatments the factors were 3.4, 2.9, and 3.3 for DS, DW, and DN procedures, respectively. While three of the treatments show similar behavior, DS deviates for high concentrations reaching a plateau, see dashed red rectangle in Fig. 5a. This means the sucrose excipient reduces the negative effect over drying NPs and their coalescence when the drug load is augmented. In the case of the DN treatment, we propose the Na^+^ and Cl^−^ ions altered the pH and the net charge on the protein surface through amino acid hydrogens interacting with different ions in solution, resulting in an acidic pH change. At pH 4.9 there is a lack of electrostatic repulsion and thus amorphous aggregates are readily formed through nonspecific interactions^3^. Additionally, the increase in capsaicin concentration generates a more hydrophobic environment, which increases the formation of aggregates. Future work may be directed towards establishing possible relations between formulation and drying processes.

In contrast to the *Ed* parameter, the shape factor (*Sf*, related to circularity) and aspect ratio (*Ar*, related to ellipticity) showed only small changes against capsaicin concentration for DW, DN, and DS treatments, see empty squares, empty circles and full triangles in Fig. 5b,c. On the other hand, samples with no drying treatment, ND, showed affectations with the concentration as can be seen in Fig. 5b,c, full circles; thus, the change of shape was related to the drying process. This means that the particles not dried show gradual changes of *Sf* and *Ar* associated to an increment of capsaicin concentration. Also, interestingly, the treatment with sucrose (DS) resulted in the shape factor closest to 1, i.e. rounder particles, and the lowest aspect ratio, i.e. less ellipticity as can be seen in the full triangles of Fig. 5b,c. This is related to less aggregation and branching as a consequence of reduction in the coalescence.

A possible explanation for our results with the sucrose excipient is that when it is incorporated into the colloidal system, it could exert pressure to reduce the surface of contact between the BSA molecules of near nanoparticles due to decrease radius of gyration (spatial expansion), thus inhibiting the unfolding of the BSA in the nanoparticles^26^, and consequently improving the hydrodynamic shape of the NPs. In contrast, the loss of circular shape for the ND treatment is attributed to the increased coalescence due to higher content of capsaicin crystals, affecting the coagulation of the BSA molecules^14^. For the DW and DN procedures, the aggregation was probably due to a change in the surface charges of amino acid, thus altering the electrostatic properties of BSA^31^. The use of sucrose to dry nanoparticles improves the pharmaceutical formulations, in which, the circular shape and elliptical shape of nanoparticles are important factors for the internalization into the cell^28,32^.

### ζ-Potential and hydrodynamic diameter of aggregates

**Figure 6:**
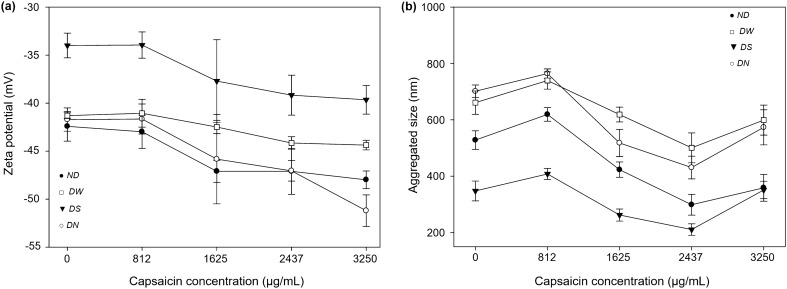
(**a**) The ζ-potential of BSA-capsaicin nanoparticles showed an increase in the electronegativity associated with drying treatments. ND, DW, and DN treatments showed a negative effect, at 3250 µg mL^−1^ the saturation of capsaicin produces a significant increment of the ζ-potential. While the DS treatment showed a low increase of electronegativity due to the controlled reorganization of the amino acids and capsaicin molecules on the surface of nanoparticles. (**b**) The size of the aggregates of BSA-capsaicin nanoparticles showed an oscillatory behavior associated with drying treatments. Not drying (ND), dried with water (DW) and dried with NaCl (DN) treatments showed a negative effect. In all treatments 812 µg mL^−1^ of capsaicin produces a significant increment of the size of aggregates. The treatment dried with sucrose (DS) showed a protective effect and reduce the increment of size. Values are means of three experimental replicates ± standard deviations.

In a previous work, we observed an increment of the ζ-potential as the drug load increased from low to medium capsaicin concentrations^10^. In this study, we found that the electric charge is affected by the drying process. In particular, while we observed somewhat similar values of the ζ-potential for the ND, DW, and DN treatments, a higher value of this parameter was found for the NPs subjected to the DS procedure for all drug concentration procedures (Fig. 6a, compare full circles, empty squares, and empty circles to full triangles). Also, we measured relatively large aggregates for the ND, DW, and DN treatments, while the DS samples presented smaller aggregates for all capsaicin concentrations (Fig. 6b, compare full circles, empty squares, and empty circles to full triangles). The increased electronegative values at 3250 µg mL^−1^ of capsaicin concentration for the ND, DW, and DN drying treatments (Fig. 6a, full circle, empty square, and empty circle) were not observed in the previous report. They are attributed to exposition of capsaicin molecules in the surface of NPs, and liberation of amino acids with negative charge on the Nernst layer of the particle. Eisele et al*.*^33^ describe that the negative charges on the surface of BSA are generated from deprotonation of the carboxyl end of acidic amino acids (glutamate and aspartate). According to Yang et al.^18^, the reorganization in some biopolymer nanoparticles was affected by the surface properties and internal bonds (H-bonds). In this study probably the molecular interaction between some amino acids and capsaicin affects the stability and morphology of nanoparticles due to the changes in hydrophilic/lipophilic balance, thus affecting the self-assembly process and even structures and morphologies of self-assembled materials. Capsaicin has the capacity to form a stable protein−ligand complex with BSA that mostly involve hydrophobic and electrostatic interactions^34^. However, this mechanism is not capable of binding great quantities of capsaicin, so probably there exists an alternative mechanism related to drug sites I and II in the BSA molecule (commonly called hydrophobic cavities) that facilitates the incorporation of hydrophobic drugs into the structure of BSA^3^. The modification of the drug sites I–II displays some effects such as the coalescence between nanoparticles, loss of circularity, aggregation, aberrant morphology and changes in surface of nanoparticles (ζ-potential). In nanoencapsulation of hydrophilic drugs (5-fluorouracil, vinorelbine tartrate and salicylic acid), the coalescence and aggregation of particles were not observed^3,5,6^. NPs dried with sucrose showed low aggregated size (351.1 ± 30.9 nm; see Fig. 6b, full triangles); this could be attributed to sucrose inhibiting an irreversible formation of aggregates^26^ because during the interaction of sucrose with the protein, the sucrose does not crystallize during vacuum drying^28^. This effect probably produces minor inter-particle contact and insulation of surface electrostatic charges allowed a controlled reorganization of capsaicin molecules with the amino acids of the nanoparticles.

### Stability of the nanoparticles after drying treatment

**Figure 7:**
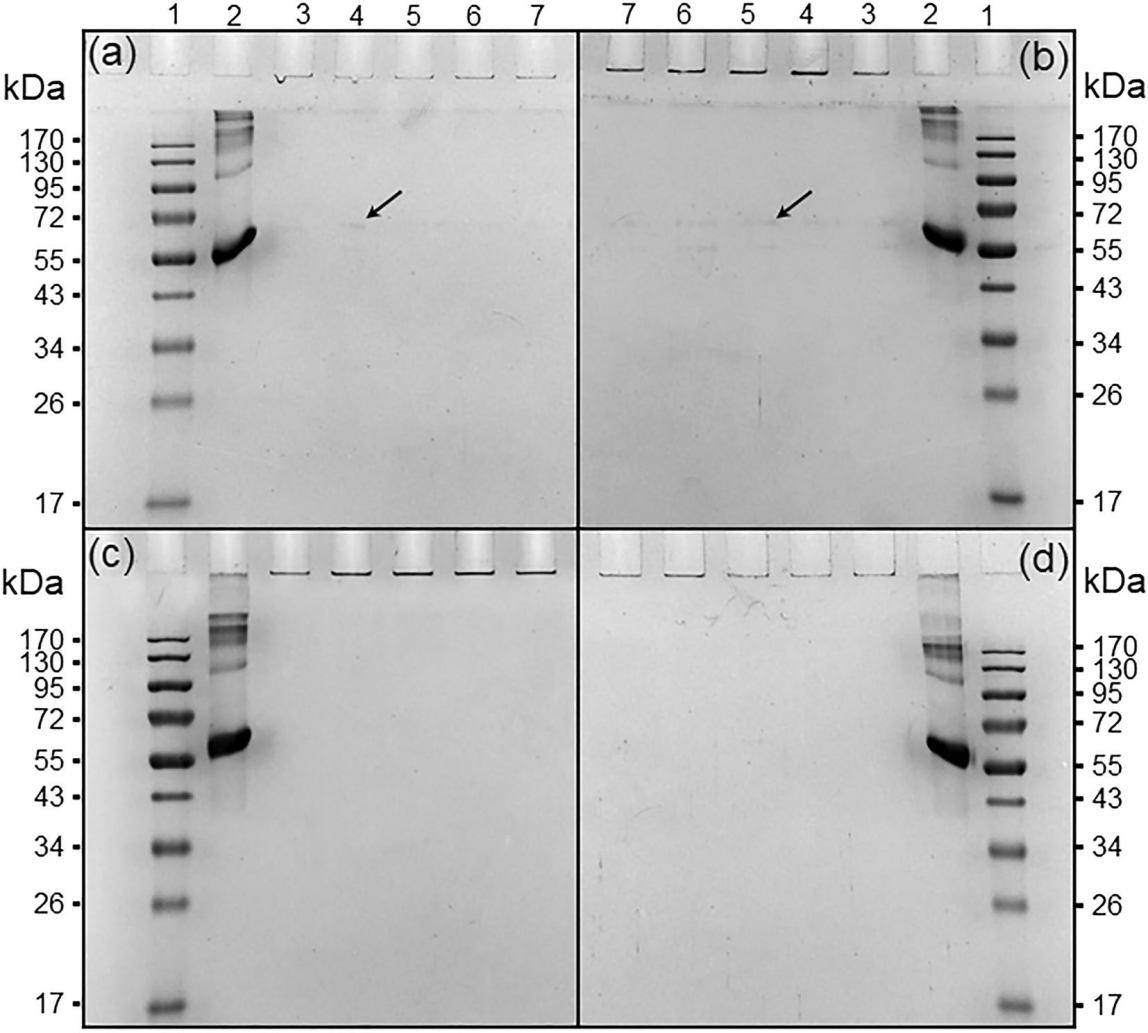
Electrophoretic patterns of disassembled molecules of BSA after nanoparticle drying treatments with several excipients and the effect of the increment of capsaicin in the nanostructuration. For all gels, lane 1 shows molecular weight MW from 10 to 170 kDa. Line 2 shows a positive control of native BSA (500 µg mL^−1^). The formulations were run on lines 3, 4, 5, 6, and 7 (nanoparticles at 0, 812, 1625, 2437, and 3250 µg mL^−1^ of capsaicin, respectively). (**a**) Treatment not drying (ND), (**b**) treatment dried with water (DW), (**c**) treatment dried with NaCl (DN), and (**d**) treatment dried with sucrose (DS).

Electrophoresis was used to determine molecular size and purity of proteins; moreover, it verified the homogeneity of the protein samples, as well as the number and molecular size of subunits^35^. In SDS-PAGE the native BSA protein migrates in response to an electrical field through pores of acrylamide gel. In this experiment, polyacrylamide gel (12% SDS-PAGE), at a constant voltage of 70 V for stacking gel and 80 V for resolving gel, allowed to determine the purity of the BSA and its molecular weight (MW). The molecular weight of native BSA was found to be 66 kDa (Fig. 7a–d, lane 2) according to the MW marker (Fig. 7a–d, lane 1). The BSA-capsaicin nanoparticles did not show migration in the acrylamide gel for any of the drying treatments, therefore, the nanoparticles were found in the loading well (Fig. 7a–d). The ND treatment at 812 µg mL^−1^ capsaicin concentration (Fig. 7a, black arrow on line 4), and DW at 1625 µg mL^−1^ (Fig. 7b, black arrow on line 5), showed a trace of free albumin at 66 kDa probably as a result of the dissembling of some albumin molecules caused by the drying process. A similar effect was observed by Wang et al.^36^, they found that the delivery of BSA from biohybrid nanoparticles increased after the exposition of NPs to excipients with low pH. The SDS-PAGE showed an intensity dependent on incubation time. This post-nanostructuration effect is excipient-time–pH-dependent. On the contrary, the DN and DS treatments showed no traces of free albumin.

Our results indicate that the BSA molecules showed high chemical stability into the structure of NPs and maintained their assemblage during the drying process. In contrast, Tarhini et al*.*^7^ found the BSA nanoparticles migrate in the gel of acrylamide and showed an intense band at 66 kDa of MW, this implied that the BSA NPs showed instability and a high degree of disassembly. Since the BSA NPs are constituted by several BSA molecules, a possible symptom of disassembly of the NPs is the presence of free albumin at 66 kDa. The orientation of the capsaicin from the core towards the surface of the NPs implies the reorganization of the hydrophobic amino acids and probably their loss during this process; in order to investigate this phenomenon, the quantification of the free amino acid was carried out.

**Figure 8:**
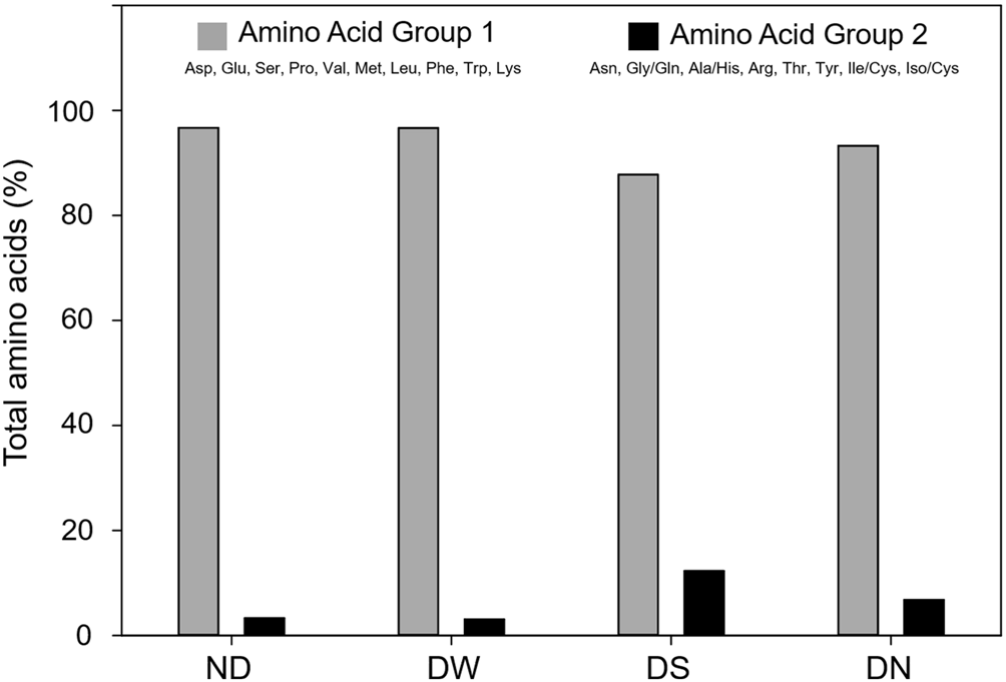
Total percentage of free amino acids quantified in ND, DW, DS, and DN treatments. The DS treatment showed the highest loss of amino acids of group 2 (GLY/GLN, ALA/HIS, ARG, THR, TYR, and Iso/CYS) involved in the mechanism of restructuration of capsaicin.

Quantification of free amino acids from dried nanoparticles with different concentrations of capsaicin allowed to establish two groups of amino acids. Group 1 includes 10 amino acids with a relevant presence in the NPs after ND, DW, DN, and DS treatments; while group 2 includes 8 amino acids with low presence, both groups are detailed in Table 2 (presence of amino acids and retention time). Notably, in group 1 serine (SER) was not observed in the ND treatment at any capsaicin concentration but was present for the other drying procedures and phenylalanine (PHE) showed irregular presence on DW, DN, and DS treatment. Regarding group 2, the DS and DN treatments showed a higher percentage of the total amino acids present in group 2, 12.27% and 6.75% respectively (see Fig. 8). The two treatments presented a larger number of amino acids of group 2, with 6 amino acids; in particular, DS had the lowest percentage of total amino acids of group 1 and the highest of group 2 (Fig. 8).

**Table 2:** Amino acids liberated during dried processes of BSA-Capsaicin nanoparticles.

Peak	Amino acid	Retention time (min)			
		ND	DW	DS	DN
**Group 1**					
1	ASP	2.618 ± 0.015	2.718 ± 0.046	2.745 ± 0.015	2.682 ± 0.056
2	GLU	2.922 ± 0.036	2.980 ± 0.177	3.121 ± 0.016	2.985 ± 0.156
3	SER	–^a^	6.265 ± 0.111	6.356 ± 0.020	6.321 ± 0.044
4	PRO	7.277 ± 0.106	7.245 ± 0.028	7.323 ± 0.059	7.323 ± 0.016
5	VAL	10.413 ± 0.125	10.343 ± 0.052	10.366 ± 0.066	10.450 ± 0.061
6	MET	11.289 ± 0.115	10.862 ± 0.021	11.000 ± 0.020	11.100 ± 0.031
7	LEU	13.027 ± 0.032	13.019 ± 0.022	13.211 ± 0.024	13.364 ± 0.026
8	PHE	14.942 ± 0.065	14.289 ± –^b^	14.514 ± –^b^	14.726 ± –^b^
9	TRP	15.977 ± 0.036	15.731 ± 0.262	16.022 ± 0.191	16.064 ± 0.034
10	LYS	16.587 ± 0.047	16.509 ± 0.265	16.866 ± 0.031	17.023 ± 0.048
**Group 2**					
11	ASN	–^a^	–^a^	–^a^	5.807 ± –^b^
12	GLY/GLN	6.230 ± 0.028	–^a^	6.636 ± 0.019	–^a^
13	ALA/HIS	6.501 ± –^b^	6.613 ± –^b^	6.904 ± 0.065	6.852 ± –^b^
14	ARG	6.767 ± –^b^	–^a^	7.025 ± 0.095	–^a^
15	THR	7.211 ± –^b^	–^a^	7.179 ± 0.076	8.533 ± 2.223
16	TYR	–^a^	–^a^	10.057 ± 0.030	10.695 ± 1.229
17	ILE/CYS	–^a^	12.82 ± –^b^	–^a^	12.769 ± 0.148
18	Iso/CYS	–^a^	12.780 ± 0.012	12.930 ± 0.064	12.878 ± 0.022

^a^Not peak was registered.

^b^One peak was registered.

Overall, sucrose was the excipient that showed the best post-drying properties; probably, this was due to the low loss of amino acids of group 1. The molecules of sucrose reduce the instability and dragging of those amino acids that had strong water-binding into the BSA protein^28^. DS had the highest percentage of group 2 amino acids, GLY/GLN, ALA/HIS, ARG, THR, TYR, and Iso/CYS, this observation could be related to the stability of capsaicin during the reorganization into the surface of the NPs. Anand et al*.*^34^ describe 14 possible interactions with amino acid of BSA, of which five are strong interactions (Tyr400–capsaicin, Asn401–capsaicin, Lys524–capsaicin, Phe506–capsaicin, and Phe550–capsaicin); it is possible that PHE, TYR, and LYS interact strongly with capsaicin resulting in increased NP stability. Our results also suggest that the dynamical formation of complexes of capsaicin-amino acid (BSA)-water affects the structural stability of the BSA nanoparticle and its capacity to reorganize hydrophobic drugs like capsaicin. To our knowledge, this is the first report that supports the secondary post-synthesis restructuration of nanoparticles and the interaction between amino acids with hydrophobic drugs.

## Conclusions

In this study, NPs of BSA were loaded with increasing concentrations of capsaicin to evaluate the effect and changes on properties of nanoparticles after drying with several excipients. We found that the nanoparticles dried with sucrose 1 mmol reduced their aggregation and the formation of isolated NPs at the highest concentrations of capsaicin (3250 µg mL^−1^) leading to an improvement of their circular shape, reducing the elongation of the aggregates and controlling their electrochemical stability. The sucrose excipient inhibited the disassembly of BSA molecules of the nanoparticles. The instability of NPs subjected to ND, DW, and DN treatments was associated to parallel extraction of molecular water with amino acids ASP, GLU, SER, PRO, VAL, MET, LEU, PHE, TRP and LYS; in contrast, the sucrose excipient reduced the loss of these amino acids. On the other hand, the second group of amino acids, GLY/GLN, ALA/HIS, ARG, THR, TYR, and Iso/CYS, are involved in the mechanism of restructuration of the capsaicin molecules towards the surface of the NPs during the drying process. According to previous studies^10,14,34^ PHE, TYR, and LYS showed strong interaction in the relation capsaicin-amino acid–water molecules during formation of BSA-capsaicin NPs. This mechanism of secondary post-synthesis nano structuration can improve the molecular stability of the particles and their capacity of entrapping hydrophobic drugs like capsaicin. The drying parameters presented in this work could be applied to several types of nanoparticles elaborated with proteins that encapsulate hydrophobic drugs, this method of conservation improves the properties of nanoparticles and enhances parameters important to pharmaceutical and agricultural industries.

## Materials and methods

### Chemicals

The biological reagents used in this study were BSA (lyophilized powder, 66 kD) (Equitech-Bio, Kerrville, TX, USA) and pharmaceutical grade capsaicin (≥ 99%) from *Capsicum spp*. (Handim Chemical Co., Ltd, Shanghai, China). The chemical reagents were glutaraldehyde (25%) (Electron Microscopy Science, Hatfield, PA, USA), sodium chloride analytical grade (Merck, Darmstandt, Germany), acetonitrile (≥ 99.9%) (Sigma-Aldrich, St Louis, MS, USA) and absolute ethanol (≥ 99.8%) (Merck, Darmstandt, Germany). Polyacrylamide gel electrophoresis components, which consisted of acrylamide (40%), tris electrophoresis purity reagent, sodium dodecyl sulfate, ammonium persulfate, and tetramethylethylenediamine (TEMED) (Bio-Rad Laboratories, Richmond, CA, USA), Coomassie brilliant blue G-250 (Sigma-Aldrich, St Louis, MS, USA) and acetic acid glacial HPLC grade (Merck, Darmstandt, Germany) for Coomassie staining solution (CBB). For derivatization of free amino acids methanol HPLC grade (Sigma-Aldrich, St Louis, MS, USA), triethylamine (Sigma-Aldrich, St Louis, MS, USA), and phenyl isothiocyanate (Sigma-Aldrich, St Louis, MS, USA) were used.

### Preparation of BSA-capsaicin nanoparticles

Nanoparticles were prepared by the desolvation technique as described by Langer et al*.*^11^ modified by Sánchez-Segura et al*.*^14^ and readjusted from Sánchez-Arreguin et al*.*^10^. Briefly, 200 mg of BSA powder were dissolved in 2 mL of 10 mmol NaCl solution, pH 9.4, and filtered through a 0.22 μm syringe filter (Sartorius, Goettingen, Germany). The solution was maintained under agitation at 200 rpm using a stirrer (Eurostar 20, IKA, Wilmington, NC, USA) for 30 min at room temperature. The formulations of increasing concentration of capsaicin were generated by the addition of 4 mL of an ethanol-capsaicin solution at 0, 812, 1625, 2437, and 3250 µg mL^−1^ for each drying treatment. The rate of addition was 1.0 mL min^−1^ at 200 rpm of stirring speed. The crosslinking process was carried out by the addition of 5 mL of 4% glutaraldehyde in a 10 mmol NaCl solution in agitation at 1000 rpm for 30 min in dark conditions.

### Protective excipients of BSA-capsaicin nanoparticles and vacuum drying

Nanoparticles of albumin have been typically dried without water or deionized water. In order to compare the effect of excipients in the drying treatment versus traditional drying with deionized water and without drying treatment (diluted in deionized water), as a negative control, we used two protective excipients, NaCl (10 mmol, pH 9.4) and sucrose 1 mmol. The samples (1000 µL) were taken from stock of NPs and washed by three cycles of centrifugation at 12,485×*g*, 10 min, at room temperature (MC-12V, DuPont, Newtown, CONN, USA), and the dispersion of the pellet was carried out in deionized water, during every cycle. After the final cycle, the first sample that was not dried was maintained in deionized water (ND), the second sample was resuspended in 1000 µL of deionized water (DW), the third in 10 mmol NaCl solution (DN), pH 9.4, and the fourth treatment was resuspended in a 1 mmol sucrose solution (DS). Samples were homogenized by shaking in a vortex mixer (Super Mixer, LAB LINE, Melrose Park, ILL, USA) for 10 min. In DW, DN and DS treatments, the samples were dried with a MAXI-dry vacuum centrifuge (Heto-Holten A/S, Alleroed, Denmark) at 30 °C, 1300 rpm. Finally, the powder was stored at − 4 °C.

### Quantification of BSA transformed in nanoparticles and encapsulated capsaicin

The quantification of encapsulated capsaicin in NPs was done by extraction with acetonitrile as described by Sganzerla et al.^37^ and modified by Sánchez-Segura et al.^14^ and Sánchez-Arreguin et al.^10^. The BSA nanoparticles yield were calculated from recovered BSA after extraction of capsaicin. While, the encapsulated efficiency (EE%) was calculated from capsaicin quantified by HPLC as described by Bhalekar et al.^38^. The entire procedure is described in Supporting Information (see S1)^10,14,37,38^.

### Fourier transform infra red spectroscopy (FTIR)

Determination of Fourier transform infrared spectra of pure capsaicin, pure BSA and BSA-capsaicin NPs dried were performed by method described by Sánchez-Arreguin et al.^10^. These experiments are explained in Supporting Information (refer to S2)^10^.

### Determination of ζ-potential, and hydrodynamic diameter of aggregates

The ζ-potential and hydrodynamic diameter of aggregates were determined by method previously reported by Sánchez-Segura et al.^14^ and modified by Sánchez-Arreguin et al*.*^10^ as explained in Supporting Information (S3)^10,14,39^.

### Transmission electron microscopy (TEM) and morphometric analysis of nanoparticles

The morphology of the resuspended nanoparticles was examined by TEM. The experimental preparation of the samples and operating conditions of the microscope were similar to those previously reported by Sánchez-Segura et al.^14^, Sánchez-Arreguin et al.^10^ and modified by Castro-González et al*.*^40^. On the other hand, the morphometric parameters were calculated with equations proposed by Syverud et al.^41^. Parakhonskiy et al*.*^32^ and Bouwman et al*.*^42^ and modified for nanoparticle description by Sánchez-Segura et al*.*^14^ and Sánchez-Arreguin et al*.*^10^. The procedure is detailed in the Supporting Information (see S4)^10,14,32,40–42^.

### Quantification of disassembled BSA by polyacrylamide-gel electrophoresis

Sodium dodecyl sulphate polyacrylamide gel electrophoresis (SDS-PAGE) was performed using the Tris–glycine buffer system of Laemmli^43^ with modifications. In addition, to estimate the protein molecular weight (MW) we used the EZ-Run pre-stained *Rec* protein ladder with bands in the range of 10–170 kDa (Fisher Scientific, Waltham, MA, USA). To reference the MW of native BSA, lyophilized BSA (66 kDa), was dissolved in deionized water to stock concentration [500 µg mL^−1^] (Equitech-Bio, Kerrville, TX, USA). From stock, the solution was adjusted to 20 µg per lane. Finally, the BSA-capsaicin nanoparticles after drying procedures were dissolved and adjusted at a concentration of 20 µg per lane. The samples were resolved on 12% SDS-PAGE at a constant voltage of 70 V for stacking and 80 V for resolving. Then, gels were washed three times with deionized water for 5 min each and were boiled for 1 min with CBB staining solution (0.025% Coomassie dye, G-250 in 10% acetic acid). Subsequently, gels were washed again with deionized water for 5 min and clarified to visualize polypeptide bands. Gels were captured (Documentation Systems Bio-Rad) at 600 dpi resolution in tagged image file (.tif) format with 1580 × 1489 pixels in grey scale. In this format, 0 was assigned to black and 255 to white in the grey scale.

### Extraction and derivatization of free amino acids from BSA-capsaicin nanoparticles

The identification and quantification of amino acids were determined according to Bidlingmeyer et al*.*^44^ with modifications. The NPs (powder) were washed with 1000 µL of deionized water and were homogenized for 15 min and sonicated for 10 min at 25 °C. The samples were centrifuged at 12,485×*g*, for 6 min, at room temperature, the supernatant was discarded, and the pellets were dried during 20 min. The derivatization of the samples was carried out by addition of 20 µL of methanol/water/triethylamine (2:2:1) solution; then, the samples were dried for 30 min at 45 °C. The samples were resuspended with 20 µL of methanol/water/triethylamine/phenyl isothiocyanate (7:1:1:1) solution and incubated for 30 min at 25 °C, subsequently, the samples were dried. Finally, they were dissolved with 200 µL of sodium acetate trihydrate 0.1 M, pH 6.5 and were stored at − 70 °C. The samples were resolved with Shimadzu ultra-fast liquid chromatography (UFLC) prominence series system (Shimadzu, Kyoto, Japan), equipped with a LC-20AD pump coupled to a DGU-20A degassing unit, a SPD-20A dual wavelength detector, a CMB-20A system controller, a SIL-20A HT auto-sampler and a CTO-20A column oven. There were employed as the components separation role. The system was controlled by LabSolution software ver. 5.87 SP1. Chromatographic separation was performed on a C_18_ column (Agilent Technologies Eclipse XDB-C18 4.6 × 150 mm, 5 µm). Separation conditions were mobile phase A: sodium acetate trihydrate 0.1 M, pH 6.5; mobile phase B: acetonitrile/water (4:1), flow rate at 0.9 mL min^−1^; column temperature 40 °C, and UV detection at 254 nm. Analytical standards were used to confirm the identity of the peaks. Calibration solution was based on a solution of 21 amino acids standard reference material LAA21, (Sigma-Aldrich, St Louis, MS, USA) containing l-alanine, l-arginine hydrochloride, l-asparagine, l-aspartic acid, l-cysteine hydrochloride, l-cystine, l-glutamic acid, l-glutamine, glycine, l-histidine hydrochloride, trans-4-hydroxy-l-isoleucine, l-leucine, l-lysine hydrochloride, l-methionine, l-phenylalanine, l-proline, l-serine, l-threonine, l-tryptophan, l-tyrosine, and l-valine. The standard samples were prepared by injecting 60 μL in real triplicates.

## Supplementary Information

Supplementary Information 1.

## References

[1] Anhorn MG, Mahler HC, Langer K (2008). Freeze drying of human serum albumin (HSA) nanoparticles with different excipients. Int. J. Pharm..

[2] Kim TH (2011). Preparation and characterization of water-soluble albumin-bound curcumin nanoparticles with improved antitumor activity. Int. J. Pharm..

[3] Bronze-Uhle ES, Costa BC, Ximenes VF, Lisboa-Filho PN (2017). Synthetic nanoparticles of bovine serum albumin with entrapped salicylic acid. Nanotechnol. Sci. Appl..

[4] Abrego G (2014). Design of nanosuspensions and freeze-dried PLGA nanoparticles as a novel approach for ophthalmic delivery of pranoprofen. J. Pharm. Sci..

[5] Maghsoudi A, Shojaosadati SA, Farahani EV (2008). 5-Fluorouracil-loaded BSA nanoparticles: Formulation optimization and in vitro release study. AAPS Pharm. Sci. Tech..

[6] Li Y (2012). A novel active targeting preparation, vinorelbine tartrate (VLBT) encapsulated by folate-conjugated bovine serum albumin (BSA) nanoparticles: Preparation, characterization and in vitro release study. Materials.

[7] Tarhini M (2018). Protein-based nanoparticle preparation via nanoprecipitation method. Materials.

[8] Ghosh DK (2018). Antimicrobial nano-zinc oxide-2S albumin protein formulation significantly inhibits growth of “*Candidatus* Liberibacter asiaticus” in planta. PLoS One.

[9] Narendrakumar G (2018). Enhancement of biocontrol potential of biocompatible bovine serum albumin (BSA) based protein nanoparticles loaded bacterial chitinase against major plant pathogenic fungi *Alternaria alternata*. Biocatal. Agric. Biotechnol..

[10] Sánchez-Arreguin A, Carriles R, Ochoa-Alejo N, López MG, Sánchez-Segura L (2019). Generation of BSA-capsaicin nanoparticles and their hormesis effect on the *Rhodotorula mucilaginosa* yeast. Molecules.

[11] Elzoghby AO, Samy WM, Elgindy NA (2012). Albumin-based nanoparticles as potential controlled release drug delivery systems. J. Control Release.

[12] Langer K (2003). Optimization of the preparation process for human serum albumin (HSA) nanoparticles. Int. J. Pharm..

[13] Rahimnejad M, Najafpour G, Bakeri G (2012). Investigation and modeling effective parameters influencing the size of BSA protein nanoparticles as colloidal carrier. Colloids. Surf. A. Physicochem. Eng. Asp..

[14] Sánchez-Segura L, Ochoa-Alejo N, Carriles R, Zavala-García LE (2018). Development of bovine serum albumin–capsaicin nanoparticles for biotechnological applications. Appl. Nanosci..

[15] Saez A, Guzman M, Molpeceres J, Aberturas MR (2000). Freeze-drying of polycaprolactone and poly(D, l-lactic-glycolic) nanoparticles induce minor particle size changes affecting the oral pharmacokinetics of loaded drugs. Eur. J. Pharm. Biopharm..

[16] Abdelwahed W, Degobert G, Stainmesse S, Fessi H (2006). Freeze-drying of nanoparticles: Formulation, process and storage considerations. Adv. Drug. Deliv. Rev..

[17] Sánchez-Segura L, Hernández-Sánchez H, Gutiérrez-López GF (2015). Food nano- and microconjugated systems: The case of albumin–capsaicin. Food Nanoscience and Nanotechnology.

[18] Yang PP, Zhao XX, Xu AP, Wang L, Wang H (2016). Reorganization of self-assembled supramolecular materials controlled by hydrogen bonding and hydrophilic–lipophilic balance. J. Mater. Chem. B.

[19] Costa RR, Custódio CA, Arias FJ, Rodríguez-Cabello JC, Mano JF (2013). Nanostructured and thermoresponsive recombinant biopolymer-based microcapsules for the delivery of active molecules. Nanomedicine.

[20] De Freitas GB (2018). Formulation, characterization, and in vitro/in vivo studies of capsaicin loaded albumin nanoparticles. Mater. Sci. Eng. C..

[21] Karimi M (2016). Albumin nanostructures as advanced drug delivery systems. Expert. Opin. Drug. Deliv..

[22] Jincheng W, Xiaoyu Z, Sihao C (2010). Preparation and properties of nanocapsulated capsaicin by complex coacervation method. Chem. Eng. Commun..

[23] Peng W (2015). Oral delivery of capsaicin using MPEG-PCL nanoparticles. Acta Pharmacol. Sin..

[24] D’Souza L, Devi P, Shridhar MP, Naik CG (2008). Use of Fourier Transform Infrared (FTIR) spectroscopy to study cadmium-induced changes in *Padina tetrastromatica* (Hauck). Anal. Chem. Insights..

[25] Martínez-Juárez VM (2004). Specific synthesis of 5,5'-dicapsaicin by cell suspension cultures of *Capsicum annuum*. var annuum (chili jalapeño chigol) and their soluble and NaCl-extracted cell wall protein fractions. J. Agric. Food Chem..

[26] Leela JSPP, Hemamalini R, Muthu S, Al-Saadi AA (2015). Spectroscopic investigation (FTIR spectrum), NBO, HOMO–LUMO energies, NLO and thermodynamic properties of 8-Methyl-N-vanillyl-6-nonenamide by DFT methods. Spectrochim. Acta A.

[27] Lee JC, Timasheff SN (1981). The stabilization of proteins by sucrose. J. Biol. Chem..

[28] Rahman IA, Vejayakumaran P, Sipaut CS, Ismail J, Chee CK (2008). Effect of the drying techniques on the morphology of silica nanoparticles synthesized via sol–gel process. Ceram. Int..

[29] Wang B, Tchessalov S, Warne NW, Pikal MJ (2009). Impact of sucrose level on storage stability of proteins in freeze-dried solids: I. correlation of protein–sugar interaction with native structure preservation. J. Pharm. Sci..

[30] Iskandar F, Gradon L, Okuyama K (2003). Control of the morphology of nanostructured particles prepared by the spray drying of a nanoparticle sol. J. Colloid Interface Sci..

[31] Ikeda S, Morris VJ (2002). Fine-stranded and particulate aggregates of heat-denatured Whey proteins visualized by atomic force microscopy. Biomacromol.

[32] Parakhonskiy B (2015). The influence of the size and aspect ratio of anisotropic, porous CaCO_3_ particles on their uptake by cells. Dig. J. Nanobiotechnol..

[33] Eisele K (2010). Fine-tuning DNA/albumin polyelectrolyte interactions to produce the efficient transfection agent cBSA-147. Biomaterials.

[34] Anand BG, Dubey K, Shekhawat DS, Kar K (2016). Capsaicin-coated silver nanoparticles inhibit amyloid fibril formation of serum albumin. Biochemistry.

[35] Gallagher SR (2006). One-dimensional SDS gel electrophoresis of proteins. Curr. Protoc. Mol. Biol..

[36] Wang L, Liu L, Dong B, Zhao H, Zhang M, Chen W, Hong Y (2017). Multi-stimuli-responsive biohybrid nanoparticles with cross-linked albumin coronae self-assembled by a polymer-protein biodynamer. Acta Biomater..

[37] Sganzerla M, Coutinho PJ, Tavares de Melo MA, Godoy TH (2014). Fast method for capsaicinoids analysis from *Capsicum chinense* fruits. Food Rest. Int..

[38] Bhalekar M, Upadhaya P, Madgulkar A (2017). Formulation and characterization of solid lipid nanoparticles for an anti-retroviral drug darunavir. Appl. Nanosci..

[39] Fan YH (2014). Utilizing zeta potential measurements to study the effective charge, membrane partitioning, and membrane permeation of the lipopeptide surfactin. Biochim. Biophys. Acta..

[40] Castro-González CG, Sánchez-Segura L, Gómez-Merino FC, Bello-Bello JJ (2019). Exposure of stevia (*Stevia rebaudiana* B.) to silver nanoparticles in vitro: Transport and accumulation. Sci. Rep..

[41] Syverud K, Chinga G, Per Olav J, Ingebjorg L, Knut W (2007). Analysis of lint particles from full-scale printing trials. Appita J..

[42] Bouwman MA, Bosma CJ, Vonk P, Wesselingh AJ, Frijlink WH (2004). Which shape factor(s) best describe granules?. Powder Technol..

[43] Laemmli UK (1970). Cleavage of structural proteins during the assembly of the head of bacteriophage T4. Nature.

[44] Bidlingmeyer BA, Cohen SA, Tarvin TL (1984). Rapid analysis of amino acids using Pre-column derivatization. Sci. Appl..

