# Supplementary material for: Post-synthesis nanostructuration of BSA-Capsaicin nanoparticles generated by sucrose excipient
Source: Sci Rep. 2021 Apr 6;11:7549. doi: 10.1038/s41598-021-87241-8 (PMC8024356; doi:10.1038/s41598-021-87241-8)
Supplement: Supplementary file 1 — Supplementary Information 1. [file 41598_2021_87241_MOESM1_ESM.docx]

**Supplementary Information**

**Post-synthesis nanostructuration of BSA-Capsaicin nanoparticles generated by sucrose excipient**

Ramón Carriles^1^, Laura E. Zavala-García^2^, Sofía Nava-Coronel^3^, Alejandro Sánchez-Arreguin^2^, Mercedes G. López^4^, Lino Sánchez-Segura^2^*

^1^ División de Fotónica, Centro de Investigaciones en Óptica, A.C, Loma del Bosque 115, León, Guanajuato, 37150, México.

^2^ Departamento de Ingeniería Genética, Centro de Investigación y de Estudios Avanzados del Instituto Politécnico Nacional. Unidad Irapuato, Km. 9.6 Libramiento Norte, Carretera Irapuato-León, Guanajuato, 36824, México.

^3^ Departamento de Ingeniería en Nanotecnología, Instituto Tecnológico Superior de Ciudad Hidalgo, Av. Ing. Carlos Rojas Gutiérrez 2120, Ciudad Hidalgo, Michoacán, 61100, México.

^4^ Departamento de Bioquímica y Biotecnología, Centro de Investigación y de Estudios Avanzados del Instituto Politécnico Nacional. Unidad Irapuato, Km. 9.6, Libramiento Norte, Carretera Irapuato-León, Guanajuato, 36824, México.

*** Corresponding author:** Lino Sánchez-Segura

**Tel:** +52 462 623 96 00, Fax: +52 462 624 58 46

e-mail address: lino.sanchez@cinvestav.mx

**Materials and Methods**

**S1. Quantification of BSA transformed in nanoparticles and encapsulated capsaicin.**

The quantification of encapsulated capsaicin in NPs was done by recovery of capsaicin in acetonitrile as described by Sganzerla *et al*.^37^ modified by Sánchez-Segura *et al*.^14^ and Sánchez-Arreguin *et al*.^10^. The previously dried NP powder was resuspended in the original volume of 1000 µL with deionized water and homogenized; subsequently, three cycles of centrifugation at 12485 × g, 6 min at room temperature were used to wash them. The dispersion of the pellet was done by shaking in a vortex mixer and homogenized by sonication (Ultrasonic equipment, PS-20A, Shenzhen Jie Tai Co., LTD Guangdong, China) at 40 KHz, for 10 min at 25°C. Volumes of 500 μL of the ND, DW, DN and DS nanoparticles were taken and centrifuged at 12485 × g, for 6 min at room temperature; the supernatant was discarded at the final step. The pellets were broken by the addition of 1000 µL acetonitrile; the samples were homogenized for 10 min and sonicated for 20 min at 30°C. The samples were centrifuged at 12485 × g, for 10 min. Finally, the supernatants were filtered through an acrodisc of 0.22 µm pore size (Sartorius, Goettingen, Germany) and directly deposited in a HPLC vials; the samples were kept at -20°C. Denaturalized protein was incubated at 30°C for 1 h in order to evaporate residual acetonitrile. Weights of samples were registered using an analytical balance (Pioneer, Ohaus Analytical Plus, Shanghai, China).

Quantification of capsaicin by HPLC was carried out by method previously reported by Sánchez-Segura *et al*.^14^ and modified by Sánchez-Arreguin *et al*.^10^. The separation of capsaicin was achieved with a HPLC (1290, Agilent, Santa Clara, CA, USA) equipped with Zorbax Eclipse Plus C18 column with 130Å pore size (1.8 μm, 2.1 mm X 50 mm) (Part number: 959757-902, Agilent), the reversed-phase consisted of water (A) and acetonitrile (B) [A:B (40:60, *v*/*v*)] in an isocratic mode at 1.0 mL/min flow rate. The absorbance of eluted material was monitored at 280 nm and ultraviolet (UV) spectra were recorded in the range of 220−350 nm with an acquisition rate of 1.25 scan/s. The calibration curves were obtained from a capsaicin standard at different concentrations (500, 1000, 5000, and 10000 µg/mL). The curve and samples were prepared by injecting 15 μL in real triplicates.

BSA nanoparticles yield and encapsulated efficiency (*EE%*) were calculated with a modification of the equations described by Bhaleka *et al.*^38^ and modified by Sánchez-Segura *et al*.^14^ and Sánchez-Arreguin *et al*.^10^. The estimation of the BSA transformed into nanoparticles was adjusted to a total volume (11 mL) recovery from the nanoparticle coacervation process. The proposed equations (1), (2) were computed for each experiment as follows:

$\text{BSA nanoparticles yield }\left( \text{\%} \right)\text{ = }\frac{\text{BSA in nanoparticle }\left( \text{mg} \right)}{\text{initial BSA }\left( \text{mg} \right)}\text{ x100}$ (1)

$\text{Encapsulated efficiency }\left( \text{\%} \right)\text{ = }\frac{\text{encapsulated capsaicin (µg)}}{\text{ initial capsaicin (µg)}}\text{ x100}$ (2)

**S2. Fourier transform infra red spectroscopy (FTIR).**

FTIR spectra was recorded using an FTIR spectrometer (Cary 660, Agilent Technologies, Santa Clara, CA, USA) with attenuated total reflectance (ATR) fit. The configuration used in the equipment was a crystal of zinc selenide (ZnSe) for scanning spectral range between 450–4500 cm^−1^ with open-cell transmission DialPath (optical path lengths 50, 100 and 250 μm) and diamond ATR accessories. Capsaicin, BSA (standard), and NPs powder previously dried with DW, DN and DS procedures were homogenized and analyzed directly; whereas NPs under the ND procedure were dried without water to homogenize the analysis with transmission DialPath plate. Sixty-four scans were recorded with a nominal resolution of 4 cm^-1^ in transmittance mode [%T], as described previously by Sánchez-Arreguin *et al*.^10^. Single-beam spectra of the samples were collected against a background of air. Three replicates of each sample were averaged per spectrum. Spectral data were decoded with spectroscopy software SpectraGryph version 1.2 (Dr. Friedrich Menges Software-Entwicklung). Spectral graphs were analyzed in Sigma Plot 12 (Systat Software, Inc) as described before^10^.

**S3. Determination of ζ-potential, and hydrodynamic diameter of aggregates.**

The ζ-potential and hydrodynamic diameter of aggregates were evaluated by dynamic light scattering (Zetasizer Nanoanalyser ZSP, Malvern Instruments Worcester, U.K.) on scattering mode at an angle of 12.8°. The zeta potential was evaluated using a 1:20 dilution factor from not dried and resuspended nanoparticles (DW, DN and DS). Deionized water allows the measurement of the surface electrical charge of nanoparticles. Data were automatically evaluated with the Smoluchowski equation in which the particle size of ≈ 100 nm is much larger than the Debye length, ≈ 1 nm^39^. Measurements were made in a folded capillary zeta cell (DTS0012, Malvern Instruments Worcester, U.K.) as described previously by Sánchez-Segura *et al*.^14^ and modified by Sánchez-Arreguin *et al*.^10^.

**S4. Transmission electron microscopy (TEM) and morphometric analysis of nanoparticles.**

The morphology of the resuspended nanoparticles was examined by TEM (Morgagni M-268, Philips/FEI, Brno, Czech Republic and Eindhoven, The Netherlands). Samples of nanoparticles (5 μL) were placed onto a 200 mesh copper grid coated with formvar/carbon (Ted Pella Inc., Redding, CA, USA) and incubated for 10 min. Samples were contrasted with 2.5 % uranyl acetate (Electron Microscopy Science Inc., Hatfield, PA, USA) and incubated for 15 min. TEM operating conditions in all experiments were 80 kV high voltage (EHT), 8900X to 9000X for low magnification (*shadow magnification*) and high magnification from 56000X to 180000X, low vacuum pressure of 5 x 10^-3^ Pa (5 x 10^-5^ Torr) as reported previously by Sánchez-Segura *et al*.^14^ and Sánchez-Arreguin *et al*.^10^. Micrographs were captured in tagged image file (.tif) format with 1376x1032 pixels in grey scale. In this format, 0 was assigned to black and 255 to white in the grey scale.

TEM micrographs were measured by DIA. 60 images of isolated nanoparticles (circular or branched) for each drying treatment (three replicates by treatment) were cropped and transferred into a new image of 1376x1032 pixels with format .tif at 8 bits of compression in white background^10,14^. Resolution of the images was 2.1 pixels/nm. The morphometric descriptor was calculated using the “shape_descriptor1u” plugin for ImageJ v.1.49p software (National Institutes of Health, Bethesda). The parameters evaluated in the particles were effective diameter (*Ed*), aspect ratio (*Ar*), and the shape factor (*Sf*). *Ed* calculates the diameter of a fictitious circular object that has the same area as the object being measured according to Syverud *et al.*^42^ equation (3). *Ar* was calculated according to Parakhonskiy *et al.*^32^ and describes the relation between the width and length of the particles. The *Ar* is a suitable description of ellipticity e.g. for “flat” particles *Ar*< 1, for circular particles *Ar*= 1, and for elliptical particles *Ar*> 1 equation (4). *Sf* or circularity is based on the projected area of the particle and the overall perimeter of the projection according to Bouwman *et al.*^42^ equation (5).

${\text{Ed}\text{=}}^{\text{2}}\text{√}\frac{\text{A}}{\text{π}}$ (3)

$\text{Ar}\text{=}\frac{\text{Majoraxis}}{\text{Minoraxis}}$ (4)

$\text{Sf}\text{=}\frac{\text{4.π.A}}{\text{P}^{\text{2}}}$ (5)

**Acrylamide SDS-page electrophoresis**

The unedited SDS-page is shown in Fig. S1. (a) Treatment not drying (ND), (b) treatment dried with water (DW), (c) treatment dried with NaCl (DN), and (d) treatment dried with sucrose (DS).

(**a**)


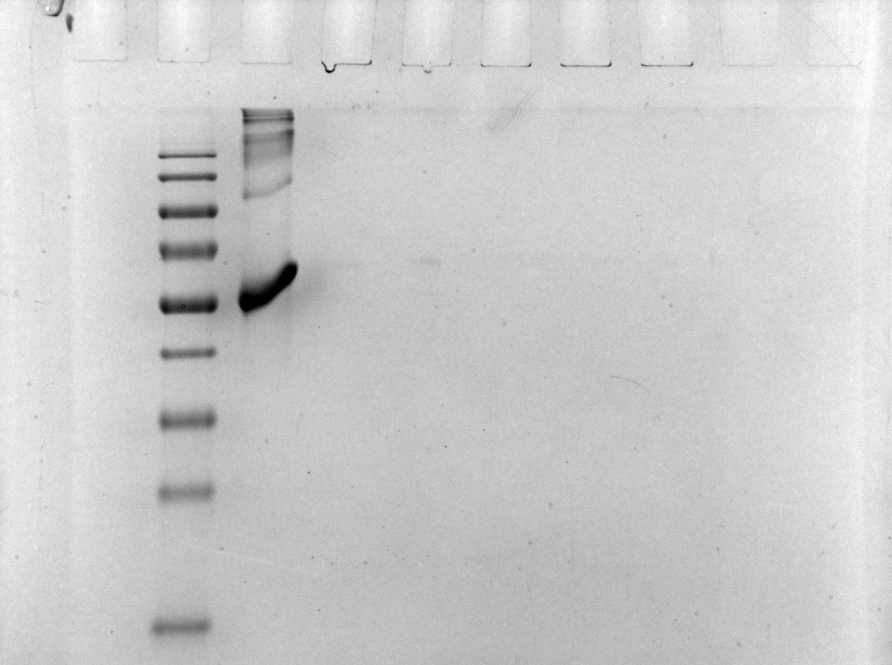
.

(**b**)


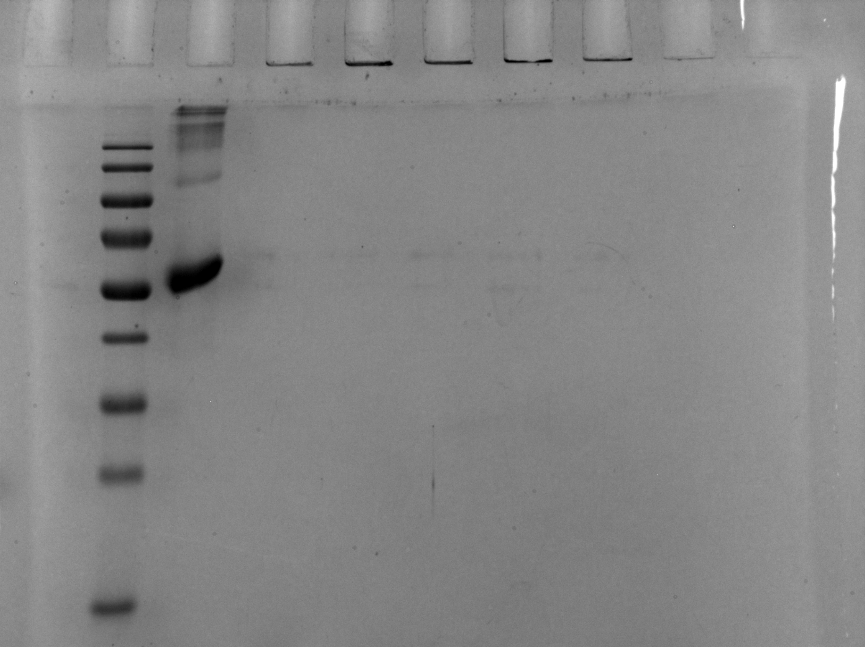


(**c**)


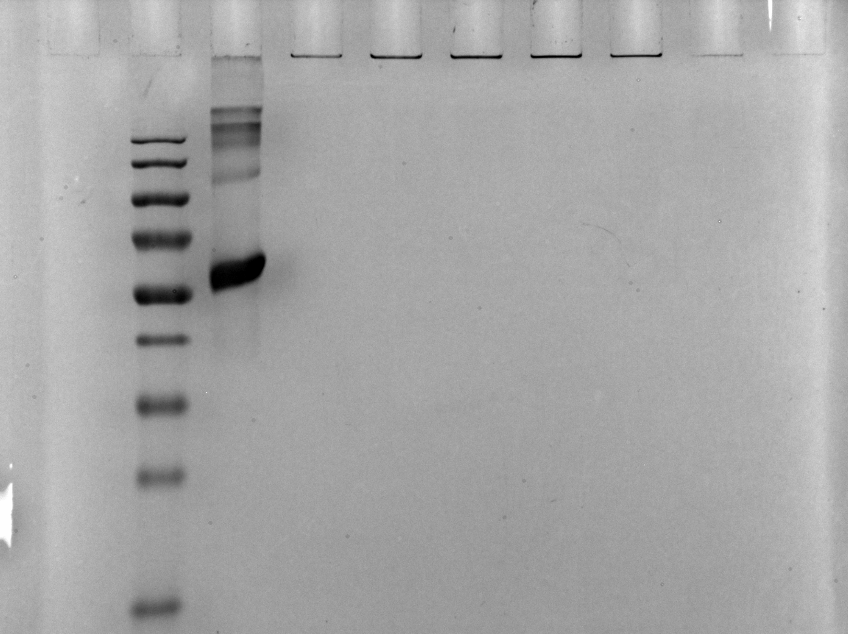


(**d**)


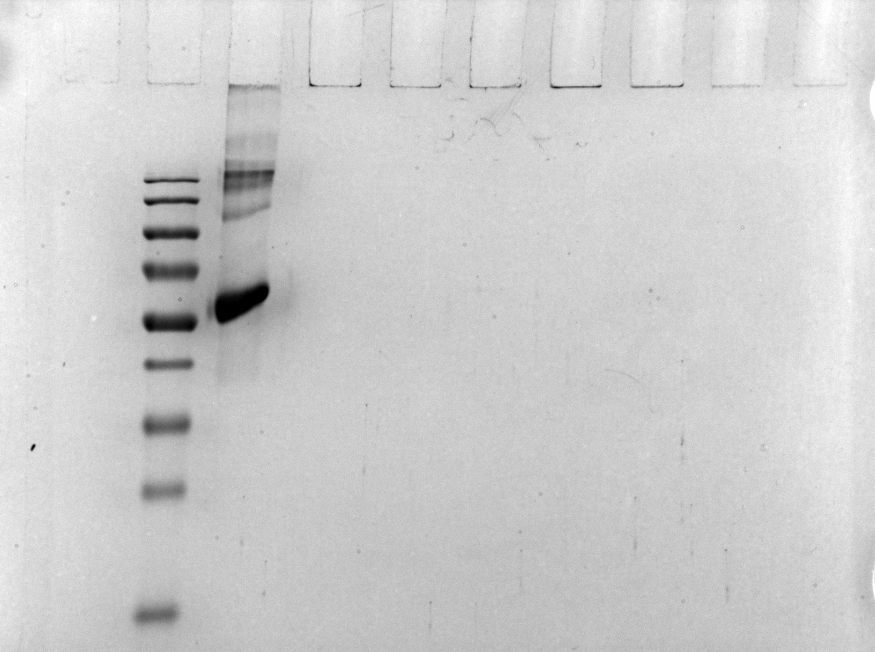


**Morphology and Morphometric Images**

Fig. S2. Morphology of BSA-capsaicin nanoparticles with different drying treatments (ND, DW, DN, DS). (a) Images without editing, (b) Binary image for Image Analysis.

(**a**)


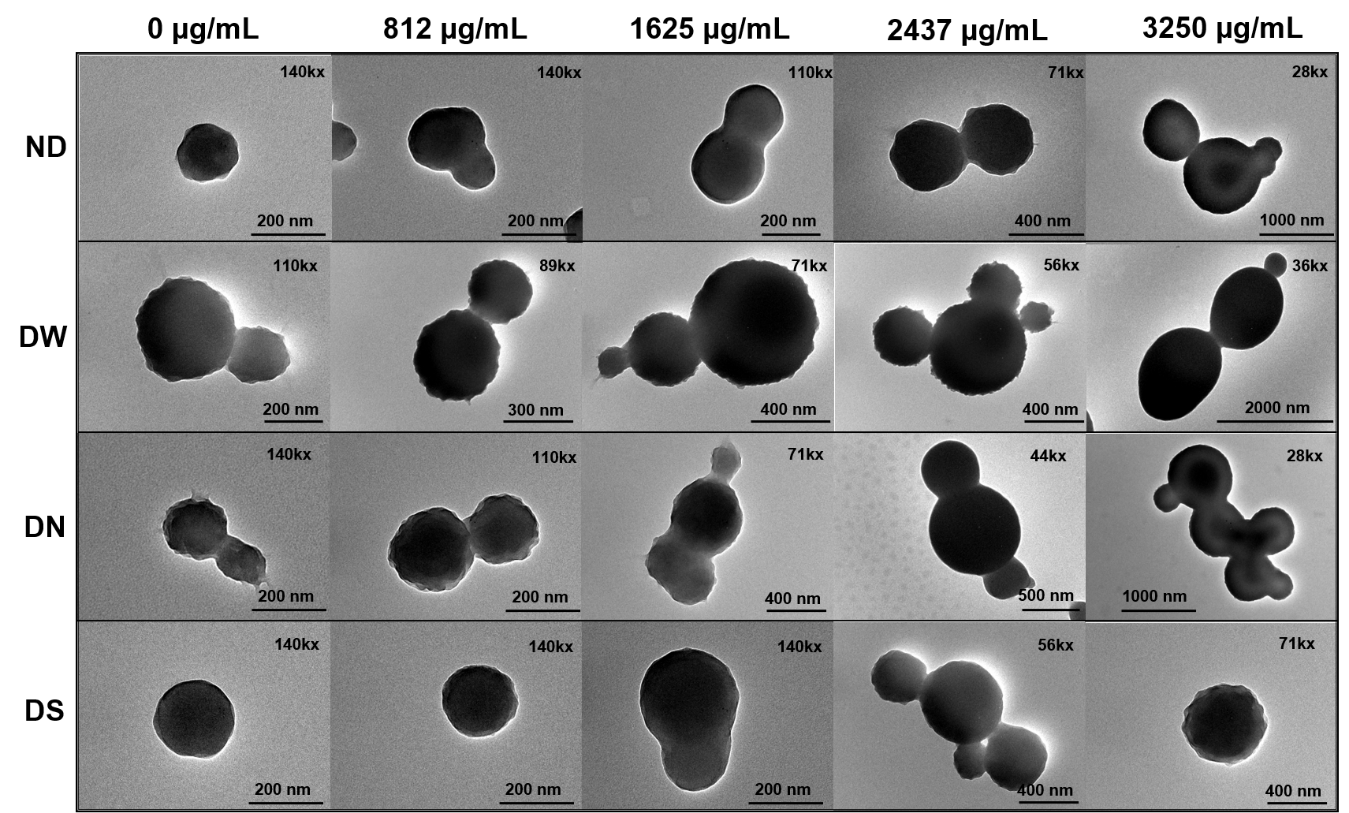


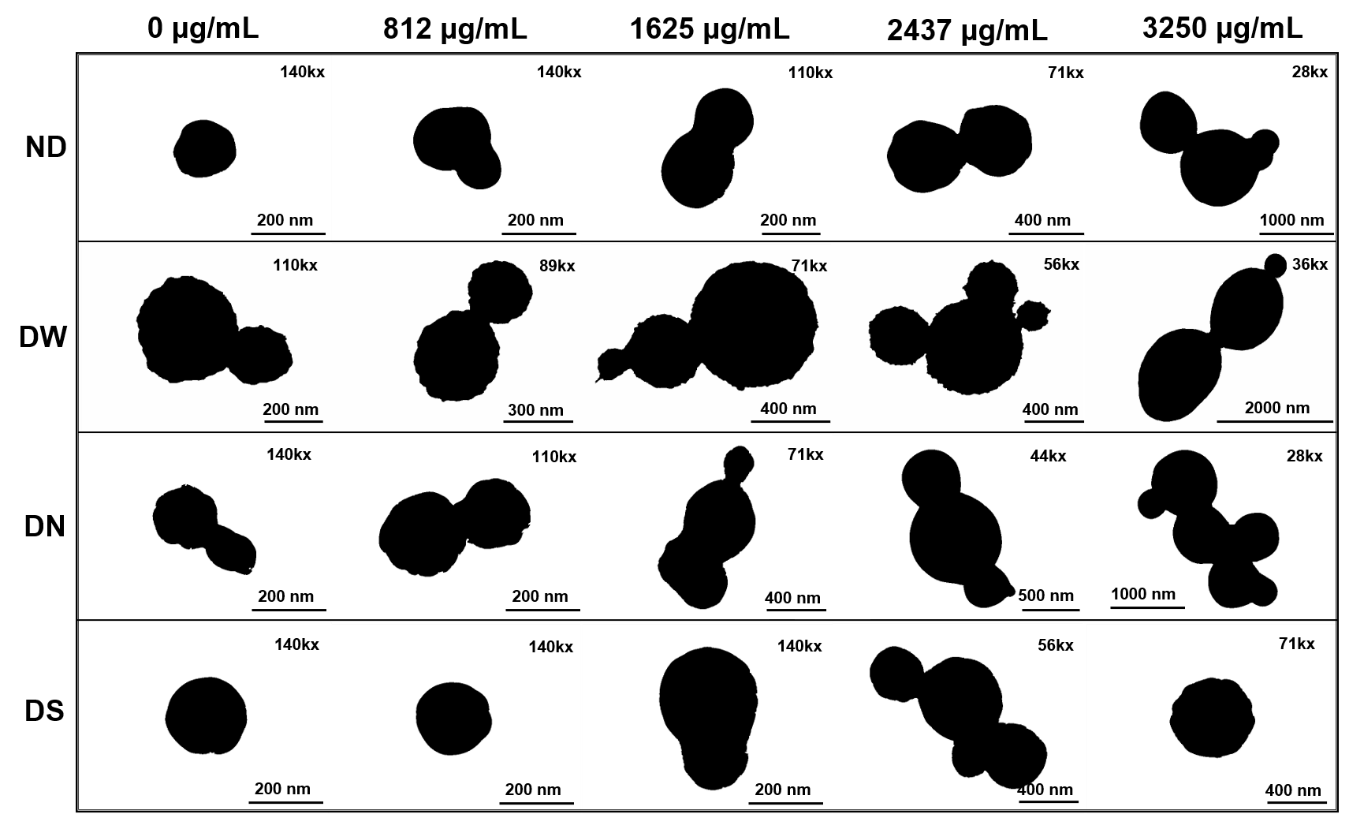


(**b**)
